# Meckel's Diverticulum in Adults: Surgical Concerns

**DOI:** 10.3389/fsurg.2018.00055

**Published:** 2018-09-03

**Authors:** Konstantinos Blouhos, Konstantinos A. Boulas, Konstantinos Tsalis, Nikolaos Barettas, Aikaterini Paraskeva, Ioannis Kariotis, Christodoulos Keskinis, Anestis Hatzigeorgiadis

**Affiliations:** ^1^Department of General Surgery, General Hospital of Drama, Drama, Greece; ^2^Fourth Surgical Department, George Papanikolaou Hospital, Medical School, Aristotle University of Thessaloniki, Thessaloniki, Greece

**Keywords:** Meckel's diverticulum, adults, complications, diverticulectomy, wedge resection

## Abstract

Since Meckel's diverticulum (MD) is rarely diagnosed in adults, there is no consensus on what type of procedure to be performed for symptomatic MD and whether to resect or not an accidentally discovered MD. Treatment of symptomatic MD is definitive surgery, including diverticulectomy, wedge, and segmental resection. The type of procedure depends on: (a) the integrity of diverticulum base and adjacent ileum; (b) the presence and location of ectopic tissue within MD. The presence of ectopic tissue cannot be accurately predicted intraoperatively by palpation and macroscopic appearance. When present, its location can be predicted based on height-to-diameter ratio. Long diverticula (height-to-diameter ratio >2) have ectopic tissue located at the body and tip, whereas short diverticula have wide distribution of ectopic tissue including the base. When indication of surgery is simple diverticulitis, diverticulectomy should be performed for long and wedge resection for short MD. When indication of surgery is complicated diverticulitis with perforated base, complicated intestinal obstruction and tumor, wedge, or segmental resection should be performed. When the indication of surgery is bleeding, wedge and segmental resection are the preferred methods for resection. Regarding management of incidentally discovered MD, routine resection is not indicated. The decision making should be based on risk factors for developing future complications, such as: (1) patient age younger than 50 years; (2) male sex; (3) diverticulum length >2 cm; and (4) ectopic or abnormal features within a diverticulum. In this case, diverticulectomy should be performed for long and wedge resection for short MD.

## Introduction

Meckel's diverticulum (MD) is the most common congenital malformation of the gastrointestinal tract involving 97% of omphalomesenteric duct malformations. It was first described in 1809 by the German anatomist Johann Meckel (1). However, it was not until almost 100 years later that the understanding of MD increased with the discovery of ectopic gastric mucosa by Salzer and associated ileum ulceration by Deetz (2). Bleeding from a MD containing ectopic gastric mucosa is the most common clinical presentation in younger patients, but it is rare in adult patients. The most common complications in adults include intestinal obstruction and diverticulitis. Due to the rarity of presentation in adults, the presence of a symptomatic MD is usually misdiagnosed preoperatively. Treatment of symptomatic MD is definitive surgery including diverticulectomy, wedge resection and segmental resection; the type of procedure depends on the integrity of diverticulum base and adjacent ileum as well as the presence and the location of ectopic tissue within MD. Prophylactic resection of an accidentally discovered MD is debatable and is reasonable that the decision-making of resection to be based on identified risk factors (3).

## Anatomy

MD is a true diverticulum involving all layers of the intestinal wall. Typically occurs within 100 cm proximal to the ileocecal valve on the anti-mesenteric border. Its blood supply is derived from the right vitelline artery which subsequently becomes the superior mesenteric artery; a mesodiverticulum may be present or not. It may have a persistent connection to the umbilicus via a fibrous band, but most often it is free and isolated (4, 5).

The lining of MD may consist entirely of intestinal mucosa, but often has ectopic mucosa. In 2005 Park et al. presented the Mayo Clinic experience of 1,476 patients with Meckel's diverticulum. Among the 180 resected diverticula in symptomatic adults, 43% contained ectopic tissue. The most common ectopic tissues were gastric (33%), pancreatic (5%), and carcinoid (2%); 63% of bleeding diverticula in adults contained ectopic gastric mucosa. Among the 806 resected diverticula in asymptomatic adults, 14% contained ectopic tissue. The most common ectopic tissues were gastric (8%), pancreatic (3%), and carcinoid (2%) (5).

Regarding the presence of ectopic tissue within MD, intraoperative identification is difficult. Park et al. showed that in 62% of MD with ectopic tissue, ectopic tissue was not palpable neither identifiable based on macroscopic wall thickening at the time of surgery (5). Regarding location of ectopic tissue, in Mukai et al. study of 8 patients with symptomatic MD containing ectopic gastric mucosa showed that long MD had ectopic gastric mucosa at the distal end and short MD in almost any area (6). In Varcoe et al. study of 25 patients with resected MD with ectopic gastric mucosa all 5 patients with long MD, defined as height-to-diameter ratio > 2, had ectopic tissue only in the diverticular tip and body, whereas 20 patients with short MD, defined as height-to-diameter ratio < 2, had wide distribution of ectopic tissue with 12 (60%) involving the whole MD including the base and eight (40%) involving the tip and body only. The authors concluded that simple transverse diverticulectomy is not recommended for short MD and macroscopic appearance in terms of height or wall thickening does not predict the presence of ectopic gastric mucosa (7).

## Complications

The total lifetime rate of complications is widely accepted at 4% with a male-to-female ratio ranging from 1.8:1–3:1 (8). Yamaguchi et al. in a retrospective study of 600 patients with MD showed the following complication rates among 287 symptomatic adult patients: (a) intestinal obstruction, 36.5%; (b) intussusception, 13.7%; (c) diverticulitis and perforation, 12.7 and 7.3% respectively; (d) hemorrhage, 11.8%; (e) tumor, 3.2% (9).

## Obstruction

MD can cause intestinal obstruction by: (a) volvulus of small intestine around the fibrous band extending from MD to umbilicus; (b) ileoileal and ileocolic intussusception; (c) incarceration of MD in an inguinal or femoral hernia known as complicated Littre's hernia; (d) axial torsion of MD with or without a fibrous band extending from MD to the mesentery or the umbilicus; (e) entrapment of small bowel beneath the blood supply of the MD known as a mesodiverticular band; (f) stricture secondary to chronic diverticulitis; (g) MD lithiasis (enterolith, bezoar); (h) entrapment of small bowel beneath a band extending from MD to the base of mesentery; (i) tumors (10).

## Diverticulitis

Inflammation of MD mimics acute appendicitis and should be considered in the differential diagnosis of a patient with right lower quadrant pain. Ludtke et al. in a retrospective study of 84 patients with MD showed that preoperative diagnosis was rare (4%); MD was a rare intraoperative finding during exploration for appendicitis with an incidence of 2.4% and a ratio of diverticulectomy to appendectomy of 1:55 (11). Diverticulitis and perforation occur at a combined rate of ~7.3%; additional complications include abscess and fistula formation (12).

## Bleeding

Lower gastrointestinal hemorrhage is the most common presentation of MD in children; the average age of presentation is 2 years, but bleeding may occur in older children and adults. Bleeding is associated with the presence of ectopic gastric and pancreatic mucosa which secretes acid and pancreatic secretions causing ulceration of the adjacent ileal mucosa. However, in adults other rare causes have been reported such as tumors (13). In adults bleeding is usually painless and presents with melena. ^99m^Tc-pertechnate is at present the investigation of choice in suspected bleeding from MD; its diagnostic sensitivity, specificity and accuracy has been reported as high as 85, 95, and 90%, respectively in the pediatric age group. Angiography may also be useful for localization of the site of bleeding and specific diagnosis (14). To date, few data are available regarding the role of capsule endoscopy (CE) and double-balloon enteroscopy (DBE) for diagnosis of MD. Krstic et al. in a retrospective study of 157 CEs for gastrointestinal bleeding showed that the diagnostic yield of CE for MD was 18.6% (15). He et al. in a retrospective study of 74 patients with surgically proven MD submitted preoperatively to CE or DBE for gastrointestinal bleeding showed that the diagnostic yield of CE and retrograde DBE for MD was 7.7 and 86.5%, respectively. Contrary to CE, the authors concluded that DBE provides a reliable diagnostic tool for patients with gastrointestinal bleeding highly suspected of having a MD (16).

## Tumors

Tumors of MD are very rare and reported only as case reports. The most common tumor is carcinoid. Park et al. in their retrospective study of 1746 patients with MD reported 4 patients (2.2%) with carcinoid, 2 patients with lipoma (1.1%) and 1 patient with leiomyosarcoma (0.6%) among 180 resected symptomatic MD in adults. The authors also reported the presence of carcinoid in 17 patients (2.1%), lipomas in 2 patients (0.2%), mucocele in 1 patient (0.1%), leiomyoma in 1 patient (0.1%), and metastatic adenocarcinoma in 1 patient (0.1%) among 806 resected asymptomatic MD in adults (5).

## Management

Treatment of symptomatic MD is definitive surgery including diverticulectomy, wedge and segmental resection performed by open or laparoscopic approach. On the contrary, routine resection of incidentally discovered MD remains controversial.

### Diverticulectomy or wedge and segmental resection for symptomatic MD?

The type of procedure to be performed for resection of symptomatic MD depends on: (a) the integrity of diverticulum base and adjacent ileum; and (b) the presence and location of ectopic tissue. As referred above, the presence of ectopic tissue cannot be accurately predicted intraoperatively by palpation and macroscopic appearance; however, when present its location can be predicted based on height-to-diameter ratio Long diverticula (height-to-diameter ratio >2) have ectopic tissue located at the body and tip, whereas short diverticula have wide distribution of ectopic tissue including the base (7). Consequently, categorization of MD in long and short, based on height-to-diameter ratio, can aid in decision making.

Based on the above, when the indication of surgery is: (1) simple diverticulitis of a long MD, diverticulectomy can be performed; (2) simple diverticulitis of a short MD, wedge resection should be performed; (3) complicated intestinal obstruction, complicated diverticulitis with inflamed or perforated base and tumor, wedge or segmental resection should be performed; (4) bleeding, wedge resection or segmental resection are the preferred methods for resection; however diverticulectomy can be performed for long diverticula (5–7). When residual ectopic tissue is histologically confirmed after simple diverticulectomy for bleeding MD, reoperation for segmental resection is necessary only after bleeding remission as simple diverticulectomy does not increase the risk of postoperative bleeding (17, 18).

Laparoscopic approach (diverticulectomy with endostaplers, wedge or segmental resection with extracorporeal or intracorporeal anastomosis) has equivalent outcomes to traditional laparotomy for symptomatic MD in both pediatric and adult patients (19). Ezekian et al. in a retrospective study of 148 cases of MD, showed that postoperative complications, rate of reoperation and readmission were similar between laparoscopy and laparotomy patients. The authors reported a conversion rate of 27.4% and suggested avoiding routine conversion for palpation of the MD or segmental small bowel resection in the absence of compelling intraoperative findings or operative complications (20).

### To resect or not an incidentally discovered MD?

Management of incidentally discovered MD remains controversial. In 2008, Zani et al. in a systematic review of 244 retrospective studies showed that resection of incidentally discovered MD had a significantly higher postoperative complication rate than leaving it *in situ*. The author's concluded that leaving an incidental discovered MD *in situ* reduces the risk of postoperative complications without increasing late complications (21). On the other hand, Zulfikaroglou et al. in a retrospective study of 76 patients with MD showed that there was no significant difference between symptomatic and asymptomatic patients with respect to postoperative complications. The authors concluded that resection of incidentally discovered MD is not associated with increased operative morbidity and mortality (22).

As management of incidentally discovered MD remains unclear, it is reasonable the decision making to be based on the presence of risk factors for developing future complications. In 2006 Robijn et al. in a systematic review suggested that decision making of resection should be based on the presence of the following risk factors: male sex, patients younger than 45 years, diverticula longer than 2 cm and the presence of a fibrous band (23). Park et al. in a retrospective study of 1476 patients with MD showed that (1) patient age younger than 50 years; (2) male sex; (3) diverticulum length >2 cm; and (4) ectopic or abnormal features within a diverticulum were all risk factors associated with development of future complications. The authors recommended removal of all incidental MD that fulfill any of these 4 criteria as when 1 criterion was met, the overall proportion of symptomatic MD was 17% and when 2, 3, and all 4 criteria were met, the proportion increased to 25, 42, and 70%, respectively (5). When resection is to be performed for an incidentally discovered MD, diverticulectomy can be performed for long diverticula and wedge or segmental resection for short diverticula (5, 6, 7).

## Conclusions

MD is the most common congenital malformation of the gastrointestinal tract. Due to the rarity of cases in adults, preoperative diagnosis may be challenging because of overlapping clinical and imaging features with other acute abdominal emergencies. Clinical manifestations arise from complications such as intestinal obstruction, diverticulitis, and bleeding. Treatment of symptomatic MD is definitive surgery, including diverticulectomy, wedge, and segmental resection. The type of procedure to be performed depends on: (a) the integrity of diverticulum base and adjacent ileum; (b) the presence and location of ectopic tissue within MD. The presence of ectopic tissue cannot be accurately predicted intraoperatively by palpation and macroscopic appearance; however, its location can be predicted based on height-to-diameter ratio as referred above (6, 7). Regarding management of incidentally discovered MD, routine resection is not indicated. The decision making should be based on risk factors for developing future complications as referred above (5, 23). Intraoperative categorization of MD in long and short is substantial for decision making of symptomatic and incidentally discovered MD. The basic principles of MD management are summarized in Table 1.

**Table 1:** Basic principles of Meckel's diverticulum management.

**Indication**	**Long diverticula**	**Short diverticula**
Simple diverticulitis	Diverticulectomy	Wedge or segmental resection
Complicated diverticulitis with inflamed or perforated base	Wedge or segmental resection	
Complicated intestinal obstruction	Wedge or segmental resection	
Bleeding	Diverticulectomy	Wedge resection or segmental resection
Incidentally discovered Meckel's diverticulum	Diverticulectomy	Wedge resection or segmental resection

## Author contributions

KAB, KB, AP, IK, NB, and CK equally contributed in writing of the article. AH and KT reviewed and had the final approval of the article.

### Conflict of interest statement

The authors declare that the research was conducted in the absence of any commercial or financial relationships that could be construed as a potential conflict of interest.

## References

[1] Meckel JF 1809 Uber die divertikel am darmkanal. Arch Physiol. (1809) 9:421–53.

[2] Choi SY Hong SS Park HJ Lee HK Shin HC Choi GC. The many faces of Meckel's diverticulum and its complications. J Med Imaging Radiat Oncol. (2017) 61:225–31. 10.1111/1754-9485.12505 27492813

[3] Lequet J Menahem B Alves A Fohlen A Mulliri A. Meckel's diverticulum in the adult. J Visc Surg. (2017) 154:253–9. 10.1016/j.jviscsurg.2017.06.006 28698005

[4] Morris G Kennedy A Jr Cochran W. Small bowel congenital anomalies: a review and update. Curr Gastroenterol Rep. (2016) 18:16–22. 10.1007/s11894-016-0490-4 26951229

[5] Park JJ Wolff BG Tollefson MK Walsh EE Larson DR Meckel diverticulum: the Mayo Clinic experience with 1476 patients (1950-2002). Ann Surg. (2005) 24:529–33. 10.1097/01.sla.0000154270.14308.5f PMC1356994 15729078

[6] Mukai M Takamatsu H Noguchi H Fukushige T Tahara H Kaji T. Does the external appearance of a Meckel's diverticulum assist in choice of the laparoscopic procedure?. Pediatr Surg Int. (2002)18:231–3. 10.1007/s003830100663 12021967

[7] Varcoe RL Wong SW Taylor CF Newstead GL. Diverticulectomy is inadequate treatment for short Meckel's diverticulum with heterotopic mucosa. ANZ J Surg. (2004) 74:869–72. 10.1111/j.1445-1433.2004.03191.x 15456435

[8] Kusomoto H Yoshida M Takahashi I Complications and diagnosis of Meckel's diverticulum in 776 patients. Am J Surg. (1992) 164:382–3. 10.1016/S0002-9610(05)80909-2 1415948

[9] Yamaguchi M Takeuchi S Awazu S Meckel's diverticulum. Investigation of 600 patients in Japanese literature. Am J Surg. (1978) 136:247–9. 10.1016/0002-9610(78)90238-6 308325

[10] Bingham JR Causey MW Haque MI. Phytobezoar within Meckel's diverticulum: an unusual cause of intestinal obstruction. Am Surg. (2014) 80:94–6. 24666857

[11] Lüdtke FE Mende V Köhler H Lepsien G Incidence and frequency or complications and management of Meckel's diverticulum. Surg Gynecol Obstet. (1989) 169:537–42. 2814770

[12] Yahchouchy EK Marano AF Etienne JC Fingerhut AL. Meckel's diverticulum. J Am Coll Surg. (2001) 192:658–62. 10.1016/S1072-7515(01)00817-1 11333103

[13] Kotha VK Khandelwal A Saboo SS Shanbhogue AK Virmani V Marginean EC. Radiologist's perspective for the Meckel's diverticulum and its complications. Br J Radiol. (2014) 87:743–9. 10.1259/bjr.20130743 24611767 PMC4075535

[14] Kotecha M Bellah R Pena AH Jaimes C Mattei P. Multimodality imaging manifestations of the Meckel diverticulum in children. Pediatr Radiol. (2012) 42:95–103. 10.1007/s00247-011-2252-7 21984316

[15] Krstic SN Martinov JB Sokic-Milutinovic AD Milosavljevic TN Krstic MN. Capsule endoscopy is useful diagnostic tool for diagnosing Meckel's diverticulum. Eur J Gastroenterol Hepatol. (2016) 28:702–7. 10.1097/MEG.0000000000000603 26854797

[16] He Q Zhang YL Xiao B Jiang B Bai Y Zhi FC. Double-balloon enteroscopy for diagnosis of Meckel's diverticulum: comparison with operative findings and capsule endoscopy. Surgery (2013) 153:549–54. 10.1016/j.surg.2012.09.012 23305600

[17] Glenn IC El-Shafy IA Bruns NE Muenks EP Duran YK Hill JA. Simple diverticulectomy is adequate for management of bleeding Meckel diverticulum. Pediatr Surg Int. (2018) 34:451–5. 10.1007/s00383-018-4239-z 29460177

[18] Robinson JR Correa H Brinkman AS Lovvorn HN. Optimizing surgical resection of the bleeding Meckel diverticulum in children. J Pediatr Surg. (2017) 52:1610–5. 10.1016/j.jpedsurg.2017.03.047 28359587 PMC5610599

[19] Hosn MA Lakis M Faraj W Khoury G Diba S. Laparoscopic approach to symptomatic meckel diverticulum in adults. JSLS. (2014) 18:e2014.00349. 10.4293/JSLS.2014.00349 25489221 PMC4254485

[20] Ezekian B Leraas HJ Englum BR Gilmore BF Reed C Fitzgerald TN. Outcomes of laparoscopic resection of Meckel's diverticulum are equivalent to open laparotomy. J Pediatr Surg. (2018) 15. 10.1016/j.jpedsurg.2018.03.010 [Epub ahead of print] 29661575

[21] Zani A Eaton S Rees CM Pierro A Incidentally detected Meckel diverticulum: to resect or not to resect? Ann Surg. (2008) 247:276–81. 10.1097/SLA.0b013e31815aaaf8 18216533

[22] Zulfikaroglu B Ozalp N Zulfikaroglu E Ozmen MM Tez M Koc M. Is incidental Meckel's diverticulum resected safely? N Z Med J. (2008) 121:39–44. 18815602

[23] Robijn J Sebrechts E Miserez M. Management of incidentally found Meckel's diverticulum a new approach: resection based on a Risk Score. Acta Chir Belg. (2006) 106:467–70. 10.1080/00015458.2006.11679933 17017710

